# Retraction: CT-based risk factors for mortality of patients with COVID-19 pneumonia in Wuhan, China: a retrospective study

**DOI:** 10.3389/fradi.2023.1330251

**Published:** 2023-11-09

**Authors:** Frontiers Editorial Office

**Affiliations:** Frontiers Media SA, Lausanne, Switzerland

A Retraction of the Original Research Article CT-based risk factors for mortality of patients with COVID-19 pneumonia in Wuhan, China: a retrospective study by Li X, Li N, Chen Z, Ye L, Zhang L, Jin D, Gao L, Liu X, Lai B, Yao J, Guo D, Zhang H, Lu L, Xiao J, Huang L, Ai F and Wang X. (2021). *Front. Radiol.* 1:661237. doi: 10.3389/fradi.2021.661237

The journal retracts the July 2021 article cited above.

Following publication, the authors contacted the Editorial Office to request the retraction of the cited article, stating that the study was not approved by the Research Ethics Commission of Wuhan Central Hospital. Furthermore, the authors stated that the data underpinning the study were incomplete and the findings reported in the article are not supported by the data. An investigation was conducted in accordance with Frontiers' policies that confirmed this; therefore, the article is retracted.

This retraction was approved by the Chief Editors of Frontiers in Radiology and the Chief Executive Editor of Frontiers. The authors agree to this retraction.

